# Production of Gluten-Free Craft Beers of High Antioxidant and Sensory Quality

**DOI:** 10.3390/foods15020379

**Published:** 2026-01-21

**Authors:** Antonietta Baiano, Teresa De Pilli, Anna Fiore

**Affiliations:** Dipartimento di Scienze Agrarie, Alimenti, Risorse Naturali e Ingegneria (DAFNE), University of Foggia, 71122 Foggia, Italy; teresa.depilli@unifg.it (T.D.P.); anna.fiore@unifg.it (A.F.)

**Keywords:** brewing, craft beer, dry hopping, gluten-free, yeast strains

## Abstract

Usually, gluten-free “beers” are produced by replacing cereals containing gluten with substitutes that do not contain it or, alternatively, through enzymatic, precipitation, and/or clarification steps. The research was aimed at increasing the concentration of antioxidant compounds and improving the sensory quality of gluten-free craft beers produced from gluten-containing raw materials according to a patented brewing method that represented the starting point of the research. The experiments were organized to evaluate the effects of original combinations of four brewing procedures (Strong, Light, Very Light, Ultra-Light—differing from each other by grains/water ratio, hops/water ratio, protein rest, and boiling time), three yeast strains (M21, K97, S33), and a possible dry hopping. The beer gluten contents ranged from <5 to 13.90 mg/L. The maximum total phenolic content (200 mg/L) was detected in beers produced by combining the Light procedure, inoculation with M21 strain, and dry hopping. The highest overall sensory quality scores (4.0) were assigned to the beers obtained through the Light and Ultra-Light procedures, fermented by M21 and S33 strains, and dry hopped. Dry hopping was the main factor capable of differentiating the beers, increasing antioxidant content and improving perlage, foam characteristics, the intensity of many olfactory and gustatory characteristics, and the overall sensory quality. The brewing procedure affected all the physico-chemical indices and most sensory characteristics, except for color, citrous and spicy flavors, sweetness, effervescence, and body. The use of different yeasts did not impart significant differences for most of the variables considered.

## 1. Introduction

Beer is the fifth most consumed beverage in the world (preceded by tea, carbonated drinks, milk, and coffee), and it is the most popular alcoholic beverage [1,2]. In 2024, global beer production amounted to 1.87 billion hectoliters [3]. In the 21st century, the growth of total beer consumption is mainly due to the increasing consumption in developing countries. Currently, the largest beer consumer (data expressed as million hectoliters) is China (~490), followed by the European Union (~359), USA (~242), Brazil (~131), and Russia (~100) [4].

Conventionally, beer is produced from malted barley and wheat even with adjuncts of rye, barley, oats, spelt, kamut, or their hybridized strains. These ingredients make beer not suitable/not safe for people who suffer from celiac disease, an inappropriate immune response to gluten ingestion that is responsible for immune-mediated injury to the small intestine of genetically predisposed people, whose estimated global prevalence is around 1% [5,6,7]. Gluten is a complex mixture of hundreds of proteins, mainly glutelins and prolamins. The latter contains peptide sequences highly resistant to gastric, pancreatic, and intestinal proteolytic digestion due to their high content in proline and glutamine. These proline-rich residues are able to create highly tight structures that can mediate the adverse immune reactions [8]. Currently, the only treatment for celiac disease is a gluten-free diet.

According to prominent regulations, the maximum gluten level to label a product as “gluten-free” is 20 mg/kg [9,10,11], a value chosen as a safety threshold to avoid the negative effects caused by a high ingestion of gluten. However, the phrase “very low gluten content” can be used for products processed to reduce their gluten content to values that do not exceed 100 mg/kg [11].

Beer gluten content varies greatly, depending on the raw materials used and the brewing process. Today, highly modified malts are used to improve fermentation, with the collateral result of reducing intact gluten protein contents. During malting and brewing, some of the barley malt proteins is hydrolyzed, and most of the gluten proteins are removed with the spent grains and with hot- and cold-breaks, thus significantly reducing gluten concentration. Clarification, mainly used to remove compounds responsible for turbidity in industrial beer, causes gluten protein and gluten peptide removal as secondary effects. However, some gluten-derived peptides can remain in the final beer [12]. Watson et al. [12] analyzed three batches of 51 Belgian barley malt beers from 24 breweries, produced according to different fermentation types and styles. They found that the gluten content was comprised between <5 ppm and >80 ppm. Wheat beers have significantly higher gluten concentrations than that produced with barley (malt) due to the use of either malted wheat as the primary grist component (weiss beers) or unmalted wheat up to 40% of the grist (blanche beer). The reason for this difference can be attributed to the low solubility of barley hordeins as compared to wheat gliadins and to the higher percentage of prolamins in wheat than in barley [13,14]. Mickowska et al. [13] performed a survey on 72 commercially available beers produced in Central Europe having gluten levels ranging from 10 to over 6000 mg/L. Around 32% samples showed gluten content < 20 mg/L, and 43% of them were in the 20–100 mg/L range. The remaining 25% beers contained higher amounts of gluten. According to Fernández-Gil et al. [15], ale beers showed greater gluten content than the lager ones.

In the last years, there is a growing demand for craft beers driven by people seeking the pleasure of unique tastes and the social recognition that arise from drinking “special” beverages, i.e., beers different from those industrially produced [1]. There is no shared definition of craft beers across the world, but there is a multiplicity of both legal definitions established by national laws and working definitions given by trade organizations [16]. However, it is generally agreed that a craft brewery produces low volumes of beer, throughout not-standardized brewing processes, often starting from traditional ingredients but also with adjuncts of non-traditional ingredients as a distinctiveness sign of the producer. Craft beer numbers (3–5% of total beer consumption in Western Europe and USA, 1% in Eastern Europe [4]) place it among niche products. Craft beer is chosen by those who recognize its higher quality, are looking for less standardized sensory characteristics than those of industrial beer, and are willing to pay a higher price.

Beer haze is due to yeast, hop, polypeptides, and complexes between polypeptides and polyphenols that have not been removed through centrifugation or filtration. Unlike industrial beers, in the craft ones, cloudiness is considered as a positive attribute and, for this reason, craft beers are generally not subjected to microfiltration processes. The Italian Law [17] establishes that microfiltration (and pasteurization) must not be applied to craft beer. Consequently, craft beers often show higher gluten protein and gluten peptide concentrations than industrial beers [12]. Fanari et al. [18] analyzed the gluten content of 25 craft beer finding that none of them were gluten-free; eight beers had gluten concentrations between 20 and 100 mg/L; and the gluten contents were comprised between 39 mg/L of a Pilsner beer and 2400 mg/L of a Weizen. Moreover, wheat-based beers contained higher levels of gluten than beers produced without wheat (malted or not) in the grist.

There are several gluten-free “beers” available on the market. Many of them are produced by replacing barley or wheat malt (and other non-malted cereals containing gluten proteins) with substitute starchy sources such as cereals without gluten proteins (rice, corn, millet), pseudo-cereals (quinoa, buckwheat), or other ingredients capable of providing fermentable sugars [6]. However, according to the Italian legislation on beer production, if only gluten-free raw materials are used, the final product cannot be defined as “beer” [17]. Furthermore, these gluten-free “beers” have organoleptic characteristics that are significantly different from those of beers produced with conventional ingredients [19]—changes that are often unwelcome to regular consumers—and greater susceptibility to qualitative decline. Techniques such as selective hydrolysis (by use of prolyl-endopeptidase from *Aspergillus niger* or peptidases from barley malt) or precipitation (by use of tannins) of gluten proteins and peptides and/or clarification steps (membrane filtration, use of silica gel, etc.) are effectively applied to produce gluten-free beers from not gluten-free ingredients [20,21]. However, in Europe, the addition of such enzymes (transglutaminase is an example) must be declared on the label [22]. Other enzymes (those extracted from germinated cereals) have activity lower than that of purified enzymes and could be damaged at ethanol concentrations higher than 2% (Scherf et al., 2018) [23]. Finally, the application of silica gel/tannins needs a subsequent filtration or centrifugation thus increasing both production costs and selling prices [19].

In this context, the European patent EP 4302612A1 [24] should be considered. It concerns a method of producing gluten-free craft beer and gluten-free spent grains, starting from cereals containing gluten and without the use of exogenous enzymes. This patent represented the starting point of the research described in these pages. The overall purpose of this work was to improve the quality characteristics (in particular, those related to the concentration of antioxidant compounds and the sensory characteristics) of gluten-free craft beers produced according to the above-mentioned patent, to create a beer suitable for people suffering from celiac disease (despite being produced from raw materials containing gluten), as pleasant as a conventional beer, in particular regarding quantity and persistence of the foam and perlage, and capable of providing good quantities of beneficial substances. An experimental plan consisting of original combinations of four brewing procedures, three yeast strains, and possible dry hopping was developed to produce gluten-free craft beers differing from each other for physico-chemical and sensory characteristics. Analyses were carried out to highlight the better combinations of independent variables able to maximize antioxidant content and positive sensory properties.

## 2. Materials and Methods

### 2.1. Materials

Barley malt (*Hordeum vulgare*) cv. Fortuna, supplied by Agroalimentare Sud (Melfi, Potenza, Italy), had the following characteristics: proteins, 10.5 ± 0.8%; Kolbach Index, 40.0 ± 3.0%; and diastatic power ≥200.00 WK. The unmalted soft wheat (*Triticum aestivum*) cv. Risciola was supplied by a local farm. This cultivar was chosen because it is a traditional variety grown in central and southern Italy since 1500. Its protein content was 12.0 ± 1.0%. Beers were produced using a grain mixture of 60% malted barley and 40% unmalted soft wheat. The dried hop cones cv. Cascade (6.7% α-acid content) were supplied by Birramia (Querceta, Lucca, Italy).

### 2.2. Brewing Trials

**Figure 1:**
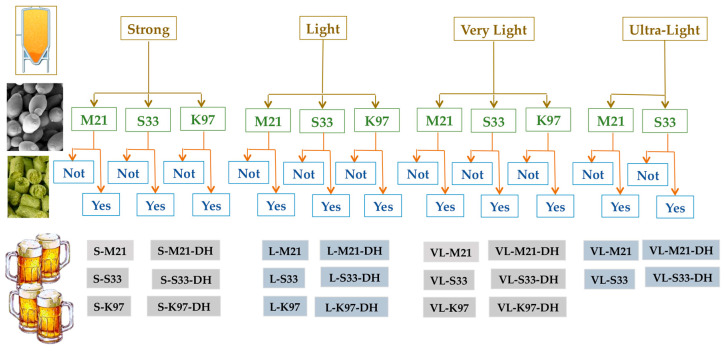
Production chart.

To produce gluten-free beers, the experiments were based on the operating conditions reported in the European patent EP 4302612A1 [24]. Briefly, the grain mixtures were placed in contact with heated water and submitted to the mashing stage that included the so-called protein rest (conditions listed in Table 1) and two other stops at temperatures between 60 and 75 °C to allow the action of β- and α-amylases. The obtained worts were then separated from the spent grains and boiled in the presence of hop cones (see Table 1 and Section 2.2.1). After cooling, the wort was fermented, possibly dry hopped, and finally bottled as described in Section 2.2.2, Section 2.2.3 and Section 2.2.4, respectively. The different brewing trials were established by combining brewing procedures, yeast strains, and dry hopping (exclusion or inclusion of this unit operation). A total of 22 different types of samples were produced (Figure 1).

#### 2.2.1. Brewing Procedures

The four different brewing procedures (i.e., combinations of operating conditions) reported in Table 1 were tested. They varied for grains/water ratio, hop cones/water ratio, duration of protein rest, and boiling time. Thirty minutes after the start of boiling, the dried hop cones (65 g/100 L) were added. The brewing trials were performed in a 50 L Braumeister system (Speidel Tank-und Behälterbau GmbH, Ofterdingen, Germany). Regardless of the highest grains/water ration of the Strong brewing, data concerning the Real Degree of Fermentation suggest a low extraction efficiency of this procedure, probably as a consequence of a poor starch conversion during mashing.

#### 2.2.2. Yeast Strains

Three *Saccharomyces cerevisiae* strains were tested:

- the top-fermenting Belgian Wit M21 strain (Mangrove Jack’s, Rosedale, Auckland, New Zealand), characterized by 70–75% attenuation, low flocculation, and ability to produce a certain balance between fruity esters and spice phenolics;
- the top-fermenting SafBrew S33 (Fermentis, Marcq-en-Barœul, France) also known as SafAle S33, characterized by 70–75% attenuation, medium flocculation, and medium production of esters and superior alcohols;
- the German ale SafAle K97 (Fermentis, Marcq-en-Barœul, France), characterized by 80–84% attenuation, low flocculation, and medium production of esters and superior alcohols.

The yeasts were inoculated according to the instructions supplied by the producers: 43.5 g/100 L, M21; 50 g/100 L, S33; and 50 g/100 L, K97. The fermentation trials were performed for 5 days at 22 °C. Then, the beers were racked, and half of them were dry hopped as described in the following section. Fermentation of both dry-hopped and not dry-hopped beers occurred for another 5 days at 20 °C.

#### 2.2.3. Dry Hopping

Half of the beers were dry hopped by infusing 300 g of Cascade hop pellets per 100 L (7.6% α-acid content, Mr. Malt, Pasian di Prato, Italy). The contact time was equal to 5 days.

#### 2.2.4. Bottling

Beers were racked, passed through a sterile gauze to retain coarse substances such as hop cones (therefore this step was not able to affect the gluten content), inoculated with the same yeast strain used for the first fermentation, added with sucrose (8 g/L), and packaged into 330 mL glass brown bottles. The overall brewing yield, which include brewhouse efficiency and packaging yield, was around 70%. This implies that 35 L of beer were packaged for each 50 L batch. The bottled beers were first conditioned at 20 ± 1 °C for 1 month and then stored at 5 ± 1 °C for 2 weeks and analyzed.

### 2.3. Analyses

The single and interactive effects of brewing procedures, inoculated yeast strain, and dry hopping (DH) on physical, chemical, and sensory quality of beers were investigated as well as their effectiveness in reducing gluten content. The 22 beer samples were coded as follows: The first term refers to the type of brewing procedure. The second term was the alphanumeric code that identifies the yeast; if present, the third term indicated that the sample was subjected to dry hopping.

#### 2.3.1. Physical and Chemical Analyses

The gluten content determination was performed through the R5 competitive Elisa Assay Method [25]. The lower limit of the quantification was 2.3 ppm (mg/kg) of gliadin, corresponding to 4.6 ppm (mg/kg) of gluten. However, although the Codex Alimentarius Commission recommends this method for testing fermented products, some limitations must be highlighted, including the following: the standardized hydrolyzed wheat prolamin calibrants do not really reflect the peptide profiles produced during fermentation since they depend on the conditions (pH, time, temperature, strains) applied. It might miss other potentially toxic gluten peptides because they are not included in the calibrants. Differences in peptide profiles can lead to gluten underestimation [26].

Color (EBC), alcohol content (%), degree of bitterness (IBU), acetic acid (mg/L), lactic acid (mg/L), total carbohydrates (g/L), and total phenolic content (TPC, mg/L) were determined through the BeerLab^®^ multiparametric analyser (CDR, Ginestra Fiorentina, Italy). The high correlations between the results obtained with CDR BeerLab^®^ and those obtained applying EBC official methods [27] have been demonstrated [28].

The pH values were measured through a BASIC 20 pH meter (CRISON, Modena, Italy). The carbon dioxide content (mg CO_2_/L) was determined though the HI 3818 Carbon Dioxide Test Kit (Hanna Instruments, Padova, Italy). The antioxidant activity (AA) was determined as described by Baiano et al. [29] and expressed as mmol Trolox Equivalent (TE)/L). Briefly, properly diluted beer samples (0.1 mL) were added to 3.9 mL of a 6 × 10^−5^ M methanol DPPH radical solutions. The absorbance at 515 nm was measured at 0 min, 1 min, and every 15 min until the reaction reached a plateau.

#### 2.3.2. Sensory Evaluation

A trained panel composed of 12 members (six men and six women), who were regular consumers of industrial and craft beer and aged between 18 and 60 years, was used to perform the quantitative descriptive analysis (QDA) of the experimental beers. The selected panelists had many years of experience, resulting from having been trained and then regularly involved in all the experimental brewing activities conducted by the research group. The sensory evaluations were performed in a room free of noise and foreign odors, illuminated with white light, and kept at 22 ± 1 °C. The serving size was equal to 50 mL, and the three digit-coded beers were presented to the panelists in crystal goblets at a temperature of 5 ± 1 °C. Each panelist assessed all beers in triplicate in the course of eight different sessions. The order of presentation of the beers was randomized across sessions. The panelists evaluated and assigned a score to the following descriptors [30]: 6 visual (color, amount, and persistence of foam; color and clarity of the liquid fraction; perlage); 8 olfactory (overall olfactory intensity [OI], olfactory elegance, i.e., a combination of balance and harmony of flavors [OE], malty, hoppy, floral, citrous, spicy, and yeasty); 4 gustatory (sweetness, bitterness, acidity/sourness, and saltiness), and 3 tactile (alcoholicity, effervescence, and body/fullness). Panelists were also asked to objectively evaluate the beer overall sensory quality (OSQ), defined as reported by Baiano et al. [31]: “The overall impression of the product deriving from the evaluation of all the sensory attributes”. The foam color (1 = white; 2 = rose; 3 = cream; or 4 = capuchin), as well as the color of the liquid fraction (1 = pale straw yellow; 2 = straw yellow; 3 = golden yellow; or 4 = amber), was evaluated on a 4-point scale. The other descriptors were evaluated on a 5-point scale.

There are three classic performance indicators. The performances of both individual panelists and the whole panel were kept under control in terms of discrimination (ability to highlight significant differences among products); agreement (ability to assign the same scores to a given attribute of a type of products); and repeatability (homogeneity between replicated evaluation of the same type of product) [32].

#### 2.3.3. Statistical Analyses

Each brewing process was carried out twice, and each analysis was repeated thrice so that the mean values and standard deviations reported in the article were calculated on six raw data. A three-way ANOVA followed by LSD test (*p* < 0.05) was applied to evaluate the effects of brewing process, yeasts, and dry hopping on each analytical parameter and the statistical significance of the differences among the obtained beers. The principal component analysis (PCA) was applied to verify relationships among the beer samples and the corresponding physical, chemical, and sensory indices.

A cluster analysis was performed through the tree clustering method, using the complete linkage rule, and the Euclidean distance measurement system to group samples based on their similarity. The statistical analyses were performed through Statistica for Windows V. 7.0. (Statsoft, Tulsa, OK, USA).

## 3. Results and Discussion

Indices on brewing trend and efficiency such as Original Extract, Apparent Extract, and Real Degree of Fermentation (a measure of the percentage of real extract that was fermented) are reported in Table 1. RDF values were in the following ranges: 29–33% for Strong beers; 28–53% for Light beers; 33–64% for Very Light beers; and 23–31% for Ultra-Light beers. Regarding the above reported brewing yield (around 70%), it is a clear index of higher brewing losses of the experimental brewing trials than those that usually occur in an amount of about 20% in a standardized brewing process [33]. The higher losses were mainly due to racking operations that, since they were conducted in the absence of a filtration step, determined the need to remove a greater quantity of cloudy liquid.

### 3.1. Gluten Content of the Experimental Beers

The analytical results (Table 2) highlighted the ability of the experimental brewing processes to reduce gluten content to such an extent that the beer can be considered gluten-free. The corresponding concentrations ranged from a maximum of 13.70–13.90 mg/L to a minimum lower than the quantification limit. Among the considered factors, only the brewing procedure highlighted a significant influence on this variable, due to the differences, among the tested procedures, in operating conditions such as grains/water ratio and duration of the protein rest that affect the starting gluten content and the wort final gluten content, respectively.

### 3.2. Influence of Brewing Procedure, Yeast Strain, and Dry Hopping on Physical and Chemical Quality of Beers

The results of the physio-chemical analyses are reported in Table 2. The pH values ranged between 4.11 and 4.52. Concerning the effects of the single parameters, the lowest pH values were detected in beers obtained through the Strong brewing procedure, fermented by *S. cerevisiae* K97, not submitted to dry hopping. The correlations between pH values and concentrations of carbon dioxide (0.55–2.00 g/L), acetic acid (114–440 mg/L), and lactic acid (684–1335 mg/L) have been calculated, giving the following results: −0.725, −0.504, and −0.201, respectively (*p* < 0.05). These results indicated that the increase in hydrogen ion concentration that occurs during fermentation is due to factors other than the accumulation of organic acids, including the significant contribution of CO_2_ [34]. These results are in line with the existing literature. With the same inoculum, the greater acidification of Strong beers is explained by their lower cells-to-glucose ratio [35] compared to other beers, as demonstrated by the wort original extract (WOE) values of the corresponding wort samples (Table 1). The higher pH of dry-hopped beers is partially determined by the presence of hop vegetative materials and is independent of hop variety ad brewing style [36,37]. The alcohol content ranged from 3.60% to 6.50%. Regarding the single effect of brewing procedure and consistently with the grains-water ratio, the highest alcohol content was detected in Strong beers while Very Light and Ultra-Light beers showed the lowest values. The alcohol content was also affected by yeast strain, with M21 and K97 responsible for the lowest and the highest values, according to the corresponding percentages of attenuation. However, the low Real Degree of Fermentation of Strong brewing together with their alcohol content lower than that expected on the basis of the grains/water ratio could represent a technical limitation of this high-grains mashing protocol. Dry hopping increased the alcohol content (together with the carbon dioxide concentration), due to both the small amount of fermentable sugar supplied by hops [38] and the “hop creep” effect, because of which, the hops’ amylases hydrolyze carbohydrates that would otherwise be unavailable for yeast metabolism [39]. The EBC value, which ranged from 7.00 to 28.00, was increased by application of the Light brewing procedure and dry hopping. The higher EBC color of Light respect to Strong beers can be attributed to the prolonged boiling time (90 min vs. 65 min) of the first ones, which promotes Maillard reactions and caramelization despite the lower wort gravity. The higher EBC color of Light respect to VL and UL beers was due to the higher grains/water ratio of the first. The highest EBC values of dry-hopped beers depended on the release of phenolic compounds from hop pellets. The same index was downplayed by application of Very Light and Ultra-Light brewing trials. Concerning the single effect of the brewing procedure on bitterness, the highest values were detected in Strong beers, due to their higher hop/water ratio. Moreover, although dry hopping is primarily used to intensify the hop aroma, it also resulted in a significant increase in the beer bitterness units in agreement with the finding of Parkin and Shellhammer [40]. Quantification of beer carbohydrates is important for several reasons: First of all, it depends on the wort sugar concentration. Furthermore, it represents an indirect measure of the sugar used by yeast; moreover, it contributes to sweetness (although in a limited manner due to the reduced concentration of mono-, di-, and tri-saccharides) and predominantly to mouthfeel due to the higher concentration of sugar with at least four glucosyl units and non-starch polysaccharides [41]. Total carbohydrates ranged from 19.70 g/L to 49.20 g/L and were influenced by brewing process, yeast strain, and dry hopping. The highest concentrations of beer carbohydrate were detected for both the Strong and Ultra-Light procedures. In the first situation, this behavior was a consequence of the highest initial grain/water ratio, which could have negatively affected the attenuation degree. Instead, in a diluted system such as that of Ultra-Light wort (grain/water ratio equal to 0.14), β- amylases are inactivated at lower temperatures than expected [42], thus leaving a higher quantity of unfermentable carbohydrates. These results are consistent with those referred to the Real Degree of Fermentation reported in Table 1. Even though they are all top-fermenting yeasts, the K97 yeast strain left the lowest quantity of unfermented sugars accordingly to its higher attenuation ability. The lowest carbohydrate residue was found in dry-hopped beers because of the already cited “hop creep” effect [39]. The residual carbohydrates of the experimental beers were generally higher than those of standard commercial beers (typically 25–35 g/L) and low-carb gluten-free alternatives (<10 g/L), presenting caloric implications for consumers and increasing the potential risk of beer spoilage.

### 3.3. Effectiveness of Brewing Procedure, Yeast Strain, and Dry Hopping in Increasing Phenolic Content and Antioxidant Activity

One of the objectives of the work was to maximize the content of compounds having antioxidant activity. Researchers consider beers with high phenolic content as beverages of high quality due to their sensory and colloidal stability, prolonged shelf life, and high nutritional value [43,44]. The total phenolic content of the experimental beers varied over a very wide range, from 97 mg/L to 206 mg/L (Table 2), with significant influence on all the considered factors. Light and Strong procedures were able to maximize the total antioxidant content. The result of the Strong beers is explained by the greatest phenolic contribution of the raw material (due to the highest grains/water and hops/water ratios). The higher phenolic content of Light beers, which had intermediate values of grains/water and hops/water ratios, depended on the greater extraction of such compounds during the prolonged boiling of the wort. In both types of beers, the extraction of polyphenols deriving from dry hopping was further improved by the higher ethanol levels [45] produced during fermentation, with respect to the other two brewing procedures. The highest and the lowest phenolic contents were highlighted in beers fermented by M21 and S33 yeast strains, respectively. The different phenolic concentration among different beers may be related to the different yeast ability to produce such phenolic compounds by yeasts and/or to their different abilities for adsorbing/releasing these compounds from the matrix [46]. The M21 strain is known to impart a phenolic character to the beers obtained from its fermentation probably because of its high ability to adsorb polyphenols on the surface during the log phase (a consequence in turn of the high mannan content of its cell wall) and to desorb them when the physiological activity of yeast cells decreases (because of the decrease in the content of mannan) [47]. Dry-hopped beers had the highest phenolic content, due to the contribution of polyphenols contained in hops pellets. The antioxidant activity showed a different trend among samples with respect to the total phenolic content (Table 2). With respect to Strong and Light beers, the lowest antioxidant activity of the Very Light ones depended on the lower grains/water and hops/water ratios. If compared with the Ultra-Light beers that had the same grains/water ratio, hops/water ratio, and boiling time, the lower antioxidant activity value of Very Light beers is explained by the shorter protein rest. In fact, at the early stage of mashing, the release and solubilization of phenolics from grains by the actions of hydrolytic enzymes and water extraction is documented [48]. The different yeasts did not exert significant effects on the antioxidant activity probably because it depends not only on the phenolic contents but also on the contribution of glutathione from yeasts and melanoidins originated by boiling [49,50]. The dry-hopped beers had the highest antioxidant activity consistently with their highest phenolic contents.

### 3.4. Influence of Brewing Procedure, Yeast Strain, and Dry Hopping on Sensory Quality of Beers

**Figure 2:**
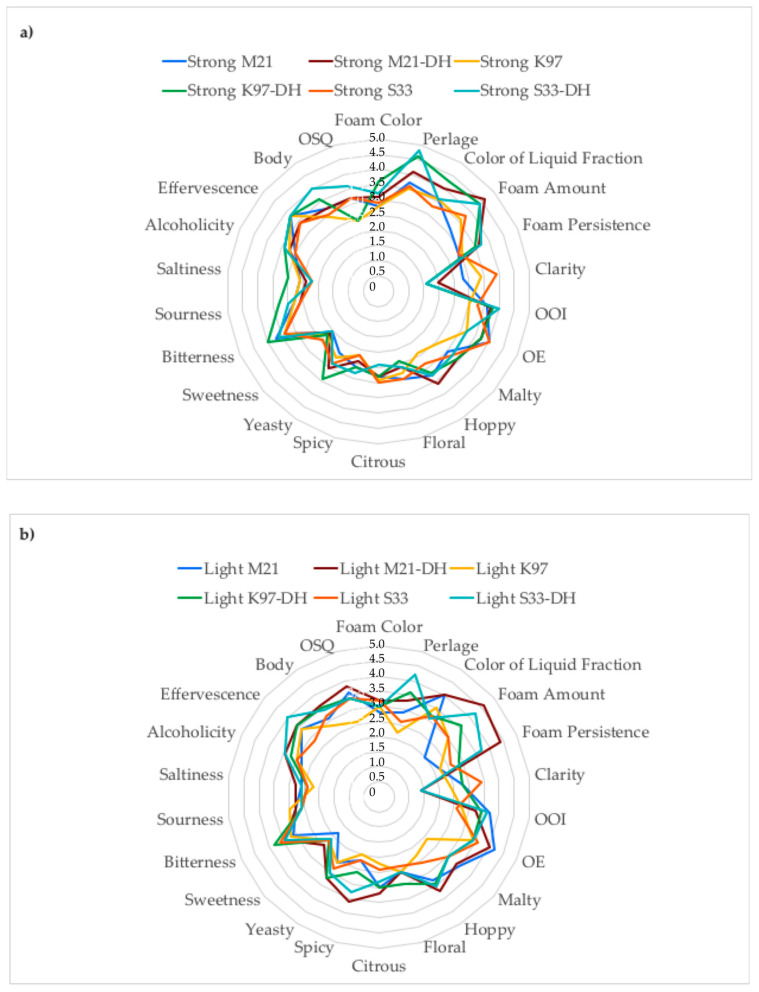

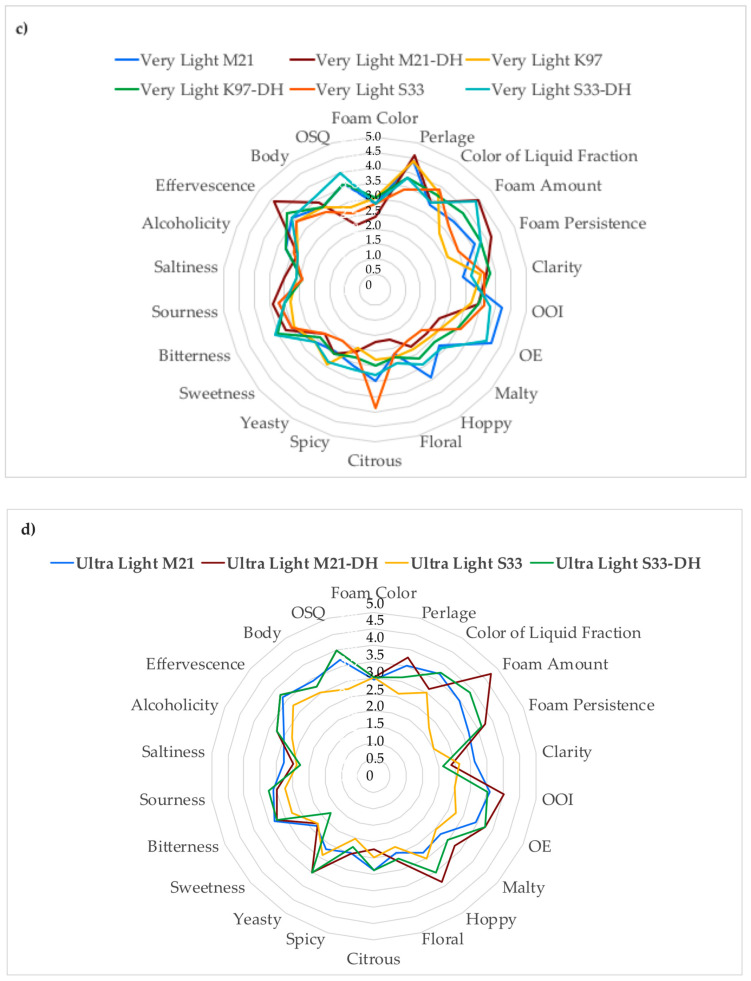
Radar diagram of the sensory characteristics of (**a**) Strong; (**b**) Light; (**c**) Very-Light; (**d**) Ultra-Light beers. DH: Dry Hopped; OOI: Overall Olfactory Intensity; OE: Olfactory Elegance; OSQ: Overall Sensory Quality.

The sensory characteristics of the experimental beers are reported in Figure 2 and Table S1. The color of the beer foam varied from 2.4 to 3.6 while that of the liquid fraction ranged from 3.0 to 4.3. However, differently from the spectrophotometric measure of beer color, its sensory evaluation was not influenced by the three considered factors (Table 3). Concerning color, the spectrophotometric approach provides superior accuracy and consistency, especially for subtle color differences as in the case of our samples. The absence of correlation between total carbohydrates and sweetness (R = 0.226, *p* < 0.05) is explained by the low residual concentration of mono- and di-saccharides [41]. The beer perlage, i.e., the formation and rising of CO_2_ bubbles from the bottom of the glass to the surface of the liquid, contributes to the beer overall sensory quality by influencing its appearance, aroma, and mouthfeel. Data of Table 3 indicate a significant dependence of perlage on brewing procedure and dry hopping. Perlage was maximized by Strong and Very Light procedures and by dry hopping. This behavior can be explained by the finding that the quantity of bubbles is positive influenced by the carbon dioxide content (higher in Strong and Very Light) and by the concentration of compounds able to reduce the surface tension such as alcohol (higher in Strong and Very Light) and iso alpha-acids (supplied by dry hopping) [51]. The same mechanisms are behind the greater amount of foam detected in Strong and dry-hopped beers and the higher persistence of foam in Very Light and dry-hopped samples. The combination of extensive proteolysis (low gluten content) and good foam stability for many of the beers produced may appear contradictory. The high concentration of proteins and surface-active species at the CO_2_/liquid interface has been generally considered as the driving force of foam stability, since it slows down drainage by lowering surface tension and increasing interface viscosity and elasticity [52,53]. Instead, recent research by Chatzigiannakis et al. [54] would indicate that the mechanisms governing foam stability depends on beer type. They confirmed that in lager beers, foam stability relies on surface viscosity while in Belgian ale beers, especially those undergoing multiple and prolonged fermentations whose viscosity is low, foam is stabilized by Marangoni stresses deriving from the presence of unconnected protein aggregates (“islands”) that remained mobile, thus impeding drainage. The observed changes in film dynamics coincide with a gradual increase in proteolysis from single to multiple fermented beers, along with the greater abundance of LTP1 associated with more intense fermentation. Beer visual clarity was affected by the presence of suspended material (peptides, vegetal material deriving from hops).

Regarding the effects of brewing process on the olfactory characteristics of beers, the Very-Light operating conditions significantly reduced the olfactory elegance and the intensity of hoppy, malty, and yeasty flavors probably as a result of the limited contribution to the aromatic fraction by both malt (low grain/water ratio, intermediate protein rest, and consequently limited production of amino-acid-derived volatile compounds) and hops (low hop/water ratios). Floral, citrous, spicy, and yeasty flavor did not show significant difference among the three yeast strains; instead, they showed a different behavior regarding the other olfactory characteristics. The M21 strain was able to enhance the overall olfactory intensity, the olfactory elegance, and the intensity of malty and hoppy flavors, while the beers fermented by K97 strains highlighted the lowest intensity of these characteristics. Concerning the influence of dry hopping on the olfactory characteristics, it was obviously able to increase the hoppy and spicy flavor, but it also enhanced the malty and yeasty flavor probably because of the biotransformation of hop-derived compounds [55]. The result was a significant increase in the overall olfactory intensity.

No gustatory and tactile characteristics were significantly influenced by the yeast strain. Moreover, the brewing procedure did not influence sweetness, effervescence, and body, and dry hopping did not significantly affect sweetness, sourness, and saltiness.

The Strong procedure maximized the perceived bitterness and alcoholicity, in agreement with the effect already highlighted on the beer bitterness units and alcohol content. The intensity of perceived sourness was minimized by the Light procedure and maximized by the Very Light procedure. However, it did not depend only on acid concentration; according to Li and Liu [56], the intensity of sour taste can be modified by the corresponding anionic acid species.

Consistently with the influence on the beer bitterness units, CO_2_ content, and alcohol content, dry hopping resulted in a significant increase in the perceived bitterness, alcoholicity, and effervescence. Moreover, hop polyphenols contribute positively to beer sensory quality by enhancing its mouthfeel [57].

The overall sensory quality, which varied in a wide range (2.8–4.0), was influenced by the three considered factors in the following ways: Concerning the brewing process, the highest scores were achieved by applying the Light and Ultra-Light procedures. Among the yeasts, the highest values were assigned to the beers fermented by M21 and S33 strains; finally, the dry-hopped beers were considered to be of higher sensory quality than the untreated ones. As can be inferred from Table 3, the variables that contributed to maximize the overall sensory quality of the different types of beer depended on the type of treatment applied. Light and Ultra-Light beers owe their sensorial quality mainly to olfactory characteristics: high elegance, malty, hoppy, floral, and yeasty for the former and high intensity of malty, hoppy, and yeasty flavors for the latter. The beers fermented by M21 and S33 were judged to be of high sensorial quality thanks to the high persistence of the foam, overall olfactory intensity, high olfactory elegance, and high malty and hoppy flavors. The sensory quality of dry-hopped beers was due to the high values of perlage, amount and persistence of foam, overall olfactory intensity, malty and hoppy flavors, bitterness, alcoholicity, effervescence, and mouthfeel.

### 3.5. Principal Component Analysis and Cluster Analysis of Beers

**Figure 3:**
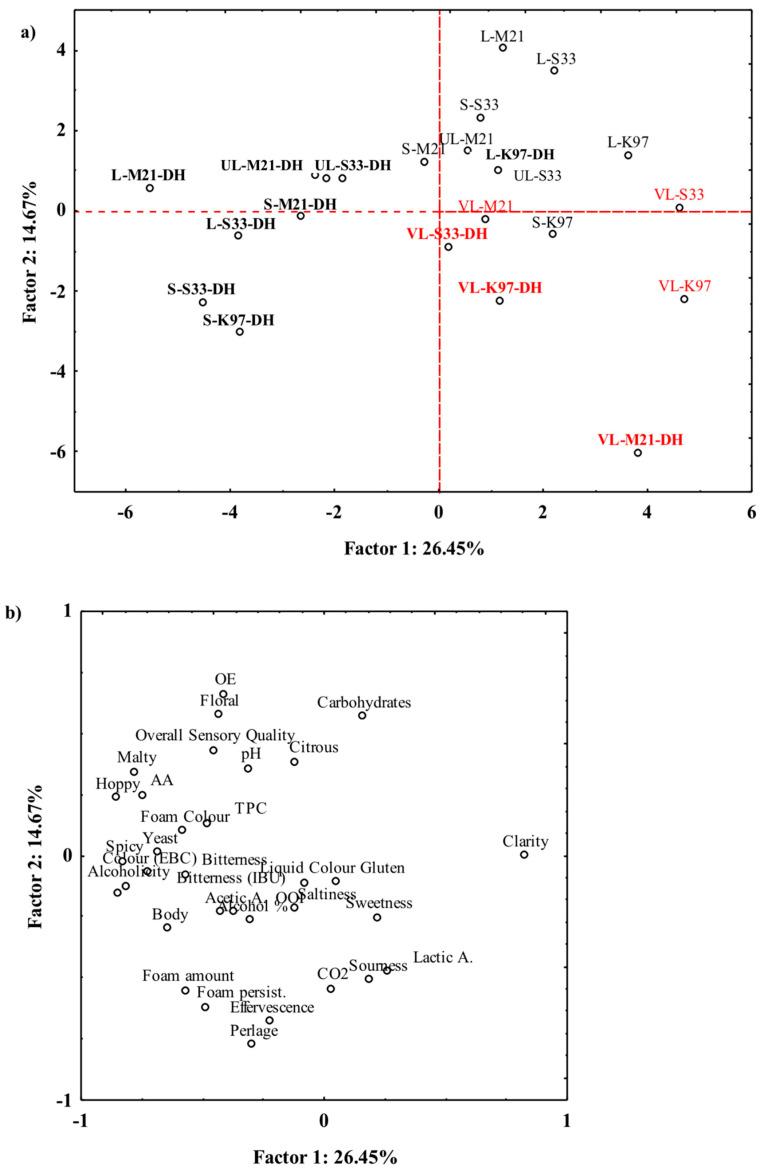
Principal component analysis applied to the physical, chemical, and sensory characteristics of beers: projection on the factorial plane of (**a**) samples and (**b**) analytical data. S: Strong; L: Light; VL: Very Light; UL: Ultra-Light; DH: Dry Hopped; OE: Olfactory Elegance; AA: Antioxidant Activity; TPC: Total Phenolic Content. Dry-hopped beers are reported in bold; Very Light beers are reported in red characters.

To highlight the relationships among beer samples and their characteristics, a principal component analysis was carried out, and the resulting graph is represented in Figure 3. As can be inferred from this representation, most of the dry-hopped beers are in the part of the plane characterized by negative values of Factor 1 while most of the samples not subjected to this treatment are in the hemi-plane identified by the positive values of the same Factor 1 (Figure 3a). The Strong dry-hopped beers fermented by S33 and K97 strains, which are placed close to each other in the quadrant characterized by negative values of both factors, shared the highest alcohol content values. Only the Very-Light samples (regardless of yeast used and any dry hopping) are grouped by brewing procedure in the quadrant characterized by positive values of Factor 1 and negative values of Factor 2 (Figure 3a). Based on the distribution of variables (Figure 3b), the higher overall sensory quality was mainly associated with higher pH values. PCA did not provide sufficient means to group beer samples since the first two factors accounted for only 41% of the variability. However, this does not mean that PCA is not suitable for the statistical treatment of our experimental data, but rather that the combinations of brewing procedures, yeast strains, and dry hopping have given rise to extremely differentiated samples.

**Figure 4:**
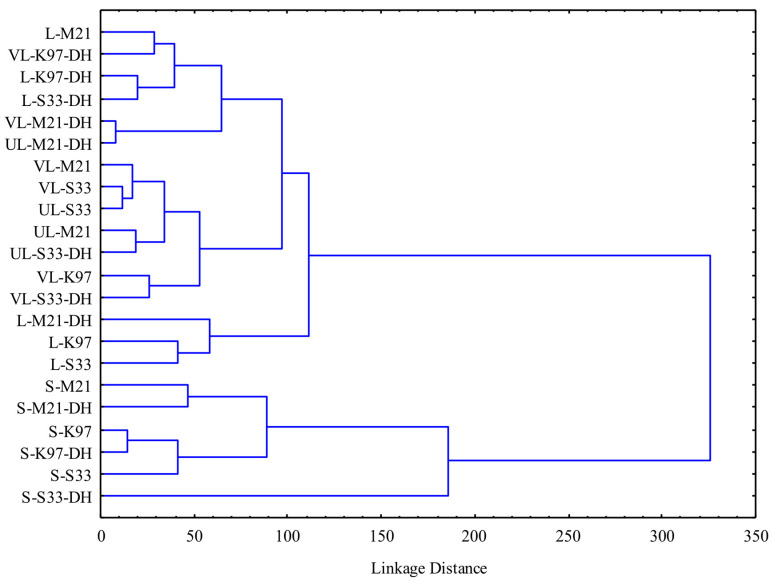
Dendrogram deriving from the cluster analysis of physical, chemical, and sensory characteristics of beers. S: Strong; L: Light; VL: Very Light; UL: Ultra-Light; DH: Dry Hopped.

At a linkage distance of 200, the cluster analysis (Figure 4) was able to differentiate two major groups of beers: Group 1 clustered the beers submitted to the Strong brewing procedures, characterized by generally higher contents of gluten, acetic acid, alcohol, remaining carbohydrates, high degree of bitterness (IBU), and, among the sensory characteristics, higher foam amount, overall olfactory intensity, and perceived bitterness, and Group 2 clustered all the other experimental beers. Whitin Group 1, at a linkage distance of 150, S-S33-DH beer stands out from all other strong beers for its lowest gluten content and intensity of citrous notes and for its highest values of the following variables: contents of CO_2_ and acetic acid and color (EBC), bitterness (IBU), antioxidant activity, perlage, foam persistence, OOI, spicy flavor, sourness, body, and OSQ. At a linkage distance of 80, three sub-clusters are recognizable within Group 2. As can be inferred by Figure 4, they have a quite heterogeneous composition, without dominant drivers of similarity, except for the brewing procedure that, to some extent, affected clustering. In fact, one sub-group was made of three Light beers; another sub-group includes four Very-Light and three Ultra-Light beers; the last sub-group includes three Light beers, two Very-Light beers, and a Ultra-Light sample. Each sub-group includes beers fermented by different yeast strains as well as dry-hopped and not dry-hopped samples.

## 4. Conclusions

The brewing procedures tested were able to produce gluten-free craft beers starting from raw materials containing gluten. By combining three factors, namely brewing procedures, yeasts, and dry hopping, it was possible to find the combination(s) able to maximize the content of antioxidants and the sensory quality of the experimental gluten-free beers. The highest total phenolic content was obtained by the combination of Light brewing, M21 yeast strain, and dry hopping. The highest OSQ values were attributed to dry-hopped beers, obtained from Very-Light wort fermented by *S. cerevisiae* S33 and Ultra-Light wort fermented by M21 and S33 strains. These results imply that, by appropriately modulating the process variables, it was possible to produce beers valuable from both a nutritional and a sensory point of view. Dry hopping was the factor capable of differentiating the highest number of beer variables, followed by the brewing procedure. The three yeasts did not cause significant differences for most of the variables considered. However, the low attenuation rates and the high residual sugar levels represent a technical limitation of the experimental protocol that need to be adjusted.

## Figures and Tables

**Table 1:** Operating conditions of the four brewing procedures applied. Values of mashing and wort pH. Original extract (WOE) of the corresponding wort, Apparent Extract (AE), and Real Degree of Fermentation (RDF) of the beers before bottling.

Operating Conditions	Brewing Procedures							
	Strong		Light		Very Light		Ultra-Light	
Grains/Water (kg/L)	0.18		0.16		0.14		0.13	
Hop cones/Water (g/L)	1.48		1.11		1.00		1.00	
Protein rest at 55 °C (min)	10		30		30		60	
Boiling (min)	65		90		90		90	
Mash pH	5.54 ± 0.02		5.58 ± 0.04		5.62 ± 0.01		5.56 ± 0	
Wort pH	5.46 ± 0.03		5.47 ± 0.03		5.56 ± 0.01		5.41 ± 0.01	
WOE (°P)	13.2 ± 0.1		11.0 ± 0.2		11.0 ± 0.1		10.5 ± 0.2	
	AE (°P)	RDF (%)	AE (°P)	RDF (%)	AE (°P)	RDF (%)	AE (°P)	RDF (%)
M21	3.5 ± 0.1	33	3.5 ± 0.1	28	3.0 ± 0.1	33	3.5 ± 0.1	27
M21-DH	3.5 ± 0.1	33	3.0 ± 0.2	33	3.0 ± 0.2	33	3.3 ± 0.2	31
K97	4.0 ± 0	29	3.0 ± 0	33	1.0 ± 0	64		
K97-DH	3.0 ± 0.1	38	3.0 ± 0	33	1.5 ± 0.1	53		
S33	3.5 ± 0	33	1.5 ± 0	53	3.0 ± 0	33	3.5 ± 0.1	27
S33-DH	3.5 ± 0.2	33	2.0 ± 0	45	3.0 ± 0	33	4.0 ± 0	23

**Table 2:** Interactive and single effects of brewing procedures, yeast strains, and dry hopping (DH) on the physical and chemical characteristics of beers. Data are expressed as mean value ± standard deviation.

Beers	Gluten (mg/L)	pH	CO_2_ (g/L)	Acetic Acid (mg/L)	Lactic Acid (mg/L)	Alcohol (%, ABV)	Color (EBC)	Bitterness (IBU)	Total Carbohydrates (g/L)	TPC (mg/L)	AA (mM TE/L)
Interactive effects of brewing *yeast* application of dry hopping											
Strong-M21	13.9 ± 0.3	4.14 ± 0.01	1.75 ± 0.10	255.00 ± 2.00	1058.00 ± 4.00	4.90 ± 0.1	12.00 ± 1.73	27.00 ± 0.05	44.60 ± 0.40	179 ± 1	1.101 ± 0.229
Strong-M21-DH	13.7 ± 0.4	4.21 ± 0.03	1.63 ± 0.10	290.00 ± 2.00	1077.00 ± 5.00	5.50 ± 0.20	14.33 ± 1.15	31.90 ± 0.05	32.40 ± 0.30	198 ± 1	1.095 ± 0.008
Strong-K97	12.8 ± 0.5	4.11 ± 0.01	1.81 ± 0.06	285.00 ± 1.00	892.00 ± 3.00	5.50 ± 0.10	8.67 ± 0.58	27.80 ± 0.10	30.70 ± 0.10	134 ± 1	0.794 ± 0.167
Strong-K97-DH	12.5 ± 0.2	4.14 ± 0	1.75 ± 0.10	290.00 ± 1.00	880.00 ± 10.00	6.50 ± 0.10	9.33 ± 0.58	31.00 ± 1.50	22.90 ± 0.10	124 ± 2	1.156 ± 0.052
Strong-S33	12.3 ± 0.1	4.14 ± 0.01	1.63 ± 0.13	320.00 ± 2.00	824.00 ± 1.00	4.90 ± 0.10	8.00 ± 0.00	32.40 ± 1.00	49.20 ± 0.90	124 ± 1	1.042 ± 0.009
Strong-S33-DH	12.0 ± 0.1	4.17 ± 0.03	2.00 ± 0.05	440.00 ± 2.00	961.00 ± 5.00	5.70 ± 0.20	18.67 ± 1.15	36.50 ± 0	34.70 ± 0.30	172 ± 1	1.190 ± 0.008
Light-M21	9.1 ± 0.2	4.50 ± 0.02	1.13 ± 0.13	205.00 ± 1.00	990.00 ± 5.00	4.70 ± 0.10	8.00 ± 0.00	22.80 ± 0.10	38.40 ± 0.60	167 ± 2	1.034 ± 0.016
Light-M21-DH	8.0 ± 0.2	4.51 ± 0.01	1.13 ± 0.13	145.00 ± 1.00	1014.00 ± 5.00	3.60 ± 0.20	28.00 ± 0	38.70 ± 0.20	31.60 ± 0.40	200 ± 5	1.156 ± 0.062
Light-K97	8.1 ± 0.05	4.29 ± 0.01	1.38 ± 0.13	115.00 ± 1.00	1134.00 ± 6.00	5.30 ± 0.15	8.67 ± 0.58	21.10 ± 0.10	28.80 ± 1.00	167 ± 1	1.023 ± 0.003
Light-K97-DH	7.8 ± 0.1	4.39 ± 0.02	1.08 ± 0.08	190.00 ± 1.00	1108.00 ± 2.00	5.90 ± 0.10	16.33 ± 0.58	28.10 ± 0.10	23.40 ± 0.10	186 ± 2	1.261 ± 0.101
Light-S33	5.8 ± 0.2	4.35 ± 0.01	0.75 ± 0.05	150.00 ± 2.00	887.00 ± 3.00	4.70 ± 0.10	8.00 ± 0.00	21.90 ± 0.20	42.80 ± 0.20	150 ± 5	0.880 ± 0.014
Light-S33-DH	<4.60	4.52 ± 0.02	1.63 ± 0.13	181.00 ± 1.00	1018.00 ± 2.00	5.50 ± 0.10	23.33 ± 0.58	30.30 ± 0.10	28.20 ± 0.60	173 ± 2	1.112 ± 0.023
Very Light-M21	10.1 ± 0.1	4.36 ± 0.03	1.25 ± 0.10	160.00 ± 2.00	1124.00 ± 2.00	4.20 ± 0.00	7.00 ± 0.10	24.60 ± 0.15	35.60 ± 0.40	114 ± 2	1.044 ± 0.077
Very Light-M21-DH	9.7 ± 0.1	4.30 ± 0.02	1.50 ± 0.10	218.00 ± 2.00	1335.00 ± 5.00	4.60 ± 0.10	8.00 ± 0.10	24.70 ± 0.10	30.60 ± 0.60	130 ± 1	0.855 ± 0.005
Very Light-K97	10.5 ± 0.2	4.11 ± 0.01	1.75 ± 0.05	128.00 ± 1.00	1154.00 ± 1.00	4.60 ± 0.15	9.00 ± 0.20	22.20 ± 0.20	19.70 ± 0.30	115 ± 3	0.548 ± 0.052
Very Light-K97-DH	9.7 ± 0.2	4.28 ± 0.01	1.75 ± 0.05	194.00 ± 1.00	1008.00 ± 2.00	5.30 ± 0.15	9.00 ± 0.10	27.10 ± 1.00	20.30 ± 0.30	148 ± 2	0.878 ± 0.014
Very Light-S33	9.4 ± 0.2	4.19 ± 0.01	1.63 ± 0.13	174.00 ± 2.00	1024.00 ± 3.00	4.20 ± 0.15	7.00 ± 0.00	23.20 ± 0.30	45.20 ± 0	115 ± 2	1.000 ± 0.108
Very Light-S33-DH	8.7 ± 0.2	4.31 ± 0.01	1.50 ± 0.05	148.00 ± 2.00	1093.00 ± 3.00	4.80 ± 0.20	7.00 ± 0.05	27.20 ± 0.20	28.70 ± 0.20	127 ± 1	0.935 ± 0.002
Ultra-Light-M21	10.5 ± 0.2	4.38 ± 0.05	0.55 ± 0.05	156.00 ± 2.00	684.00 ± 3.00	4.40 ± 0.20	7.00 ± 0.05	28.20 ± 0.40	40.20 ± 0.20	97 ± 1	1.032 ± 0.024
Ultra-Light-M21-DH	10.2 ± 0.3	4.46 ± 0.03	0.74 ± 0.01	219.00 ± 3.00	772.00 ± 2.00	4.60 ± 0.20	10.00 ± 0.05	30.00 ± 0.06	34.20 ± 0.40	130 ± 1	1.088 ± 0.021
Ultra-Light-S33	11.7 ± 0.1	4.46 ± 0.04	0.87 ± 0.03	172.00 ± 2.00	742.00 ± 2.00	4.30 ± 0.20	8.00 ± 0.05	24.30 ± 0.30	36.60 ± 0.60	108 ± 1	1.009 ± 0.021
Ultra-Light-S33-DH	11.4 ± 0.5	4.45 ± 0.02	0.80 ± 0.02	144.00 ± 2.00	742.00 ± 3.00	4.80 ± 0.20	8.00 ± 0.05	28.70 ± 0.20	32.30 ± 0.21	109 ± 1	1.073 ± 0.355
Single effects of brewing											
Strong	12.87c	4.15a	1.76d	313.33c	948.67b	5.50c	11.83b	31.10c	35.75c	156.5d	1.030b
Light	7.46a	4.43c	1.18b	164.33a	1025.17c	4.95b	15.39c	27.15b	32.20b	173.8d	1.078b
Very Light	9.69b	4.26b	1.56c	170.33b	1123.00d	4.62a	7.83a	24.84a	30.02a	124.8b	0.891a
Ultra-Light	10.96b	4.44c	0.74a	172.75b	735.00a	4.52a	8.25a	27.8b	35.82c	111.0a	1.050b
Single effects of yeast											
M21	10.74a	4.36b	1.13a	206.00b	1006.75b	4.56a	11.79a	28.49a	35.95b	152.9c	0.998a
K97	10.29a	4.22a	1.58c	200.33a	1029.33c	5.52c	10.12a	26.22a	24.30a	145.7b	1.024a
S33	10.20a	4.33b	1.35b	216.13c	911.38a	4.86b	11.00a	28.06a	37.21b	134.7a	1.030a
Single effects of dry hopping											
DH not performed	10.39a	4.28a	1.32a	192.73a	955.73a	4.70a	8.30a	25.05a	37.44b	133.8a	0.96a
DH performed	10.45a	4.34b	1.41b	223.55b	1000.73b	5.16b	13.82b	30.38b	29.03a	155.00b	1.070b

In column, different letters indicate significant differences among samples (*p* < 0.05).

**Table 3:** Effects of brewing procedures, yeast strains, and dry hopping on the sensory quality of beers. Data are expressed as mean values. OOI: Overall Olfactory Intensity; OE: Olfactory Elegance; OSQ: Overall Sensory Quality.

Beers	Foam Color	Perlage	Color of Liquid Fraction	Foam Amount	Foam Persistence	Clarity	OOI	OE	Malty	Hoppy	Floral	Citrous	Spicy	Yeasty	Sweetness	Bitterness	Sourness	Saltiness	Alcoholicity	Effervescence	Body	OSQ
Single effects of brewing																						
Strong	3.1a	4.0b	3.7a	4.0c	3.3b	2.5a	3.6b	3.6ab	3.1b	3.1b	2.7b	2.8a	2.5a	2.8ab	2.1a	3.6c	2.8ab	2.5ab	3.2b	3.7a	3.3a	3.0a
Light	3.0a	3.1a	3.5a	3.4a	3.0a	2.4a	3.5b	3.7b	3.0b	3.1b	2.7b	2.8a	2.6a	2.9b	2.1a	3.4bc	2.7a	2.5ab	3.1ab	3.5a	3.3a	3.4b
Very Light	2.8a	4.1b	3.6a	3.7b	3.5c	3.4b	3.2a	3.2a	2.5a	2.6a	2.2a	2.7a	2.3a	2.5a	2.4a	3.2ab	3.1b	2.6b	2.9a	3.7a	3.3a	3.1a
Ultra-Light	3.0a	3.0a	3.3a	3.3a	2.9a	2.2a	3.3ab	3.5ab	3.0b	3.4b	2.5ab	2.5a	2.3a	3.2b	2.2a	3.1a	3.0ab	2.4a	3.0ab	3.5a	3.1a	3.5b
Single effects of yeast																						
M21	2.9a	3.7a	3.6a	3.7a	3.4b	2.5a	3.6b	3.7b	3.1b	3.3b	2.5a	2.7a	2.5a	2.8a	2.2a	3.3a	2.9a	2.6a	3.0a	3.6a	3.2a	3.3b
K97	3.1a	3.7a	3.7a	3.5a	3.0a	2.9a	3.3a	3.2a	2.7a	2.7a	2.6a	2.6a	2.3a	2.9a	2.3a	3.5a	2.9a	2.5a	3.1a	3.6a	3.2a	2.9a
S33	3.0a	3.5a	3.4a	3.6a	3.2ab	2.7a	3.4ab	3.5ab	2.9ab	3.0a	2.5a	2.8a	2.5a	2.8a	2.2a	3.3a	2.9a	2.4a	3.1a	3.5a	3.3a	3.4b
Single effects of dry hopping																						
DH not performed	2.9a	3.3a	3.5a	3.1a	2.7a	3.1b	3.3a	3.5a	2.7a	2.7a	2.5a	2.8a	2.2a	2.6a	2.3a	3.2a	2.9a	2.5a	3.0a	3.4a	3.1a	3.1a
DH performed	3.0a	4.0b	3.6a	4.3b	3.7b	2.4a	3.6b	3.5a	3.0b	3.3b	2.5a	2.6a	2.7b	3.0b	2.2a	3.5b	3.0a	2.6a	3.2b	3.8b	3.5b	3.4b

In column, different letters indicate significant differences among samples (*p* < 0.05).

## Data Availability

The original contributions presented in this study are included in the article/Supplementary Material. Further inquiries can be directed to the corresponding author.

## Supplementary Materials

The following supporting information can be downloaded at https://www.mdpi.com/article/10.3390/foods15020379/s1, Table S1. Interactive effects of brewing procedures, yeast strains, and dry hopping on the sensory quality of beers. Data are expressed as mean values ± standard deviations. OOI: Overall Olfactory Intensity; OE: Olfactory Elegance; OSQ: Overall Sensory Quality.
