# Supplementary material for: Production of Gluten-Free Craft Beers of High Antioxidant and Sensory Quality
Source: Foods. 2026 Jan 21;15(2):379. doi: 10.3390/foods15020379 (PMC12841363; doi:10.3390/foods15020379)
Supplement: Supplementary file 1 [file foods-15-00379-s001.zip › foods-4082723-supplementary.pdf]

Table S1. Interactive effects of brewing procedures, yeast strains, and dry hopping on the sensory quality of beers. Data are expressed as mean values±standard deviations. OOI: Overall Olfactory Intensity; OE: Olfactory Elegance; OSQ: Overall Sensory Quality

Beers	Foam Colour	Perlage	Colour of liquid fraction	Foam Amount	Foam Persistence	Clarity	OOI	OE	Malty	Hoppy	Floral	Citrous	Spicy	Yeasty	Sweetness	Bitterness	Sourness	Saltiness	Alcoholicity	Effervescence	Body	OSQ
**Interactive effects of brewing*yeast*application of dry hopping**																						
Strong-M21	2.8±0.4	3.7±0.5	3.6±0.5	3.1±0.7	2.9±0.5	2.8±0.8	3.6±0.9	4.0±0.7	3.0±0.0	3.3±0.7	3.0±0.7	2.8±0.8	2.6±0.5	2.4±0.5	2.0±0.7	3.7±0.4	2.8±0.4	2.6±0.7	3.0±0.7	3.8±0.4	3.2±0.4	3.2±0.4
Strong-M21-DH	3.1±0.2	4.1±0.8	4.0±0.8	4.6±0.5	3.6±1.5	2.0±0.7	3.7±0.4	3.7±0.8	3.4±0.5	3.6±0.5	2.6±0.9	2.8±0.8	2.4±0.5	3.0±1.0	2.1±0.7	3.4±0.5	2.6±0.5	2.4±0.9	3.2±0.4	3.4±0.5	3.2±0.4	3.2±0.6
Strong-K97	2.8±0.6	3.5±0.7	3.6±0.5	3.6±0.8	3.0±0.7	3.4±0.5	3.0±0.7	3.2±0.8	2.6±0.5	2.4±0.5	2.8±0.8	2.9±0.7	2.2±0.8	2.6±0.5	2.2±0.4	3.4±0.5	2.8±0.8	2.6±0.9	3.2±0.4	3.8±0.8	2.9±1.0	2.4±0.7
Strong-K97-DH	3.6±0.5	4.6±0.5	4.3±0.7	4.4±0.9	3.5±0.8	1.6±0.5	3.8±0.4	3.7±0.7	3.4±0.5	3.2±0.4	2.4±0.5	2.8±0.7	2.6±0.7	3.4±0.5	2.2±0.7	4.0±0.0	3.3±0.7	3.0±0.6	3.4±0.5	3.8±0.8	3.6±0.5	2.4±0.5
Strong-S33	2.9±0.6	3.6±0.7	3.3±0.4	3.8±0.4	2.9±0.7	3.9±0.5	3.3±0.8	4.0±0.7	3.2±0.4	2.8±0.4	3.0±0.7	3.0±0.7	2.2±0.8	2.8±0.4	2.4±0.9	3.4±0.5	2.6±0.5	2.2±0.6	3.0±0.0	3.4±0.5	3.0±0.0	3.2±0.4
Strong-S33-DH	3.2±0.7	4.8±0.5	3.6±0.7	4.4±0.9	3.7±0.4	1.6±0.5	4.0±0.7	3.2±0.7	3.2±0.4	3.3±0.4	2.6±0.9	2.4±0.5	2.8±0.8	2.8±0.4	2.0±0.7	3.6±0.5	3.0±0.7	2.2±0.8	3.4±0.5	3.8±0.4	4.0±0.0	3.6±0.9
Light-M21	2.8±0.6	2.9±0.5	4.0±0.6	2.0±0.0	2.2±0.4	2.8±0.8	3.7±0.4	4.2±0.4	3.5±0.5	3.3±0.4	2.6±0.9	3.0±0.7	2.2±0.8	2.6±0.5	1.8±0.8	3.1±0.5	2.8±0.8	2.6±0.9	2.8±0.4	3.4±0.5	3.1±0.2	3.6±0.5
Light-M21-DH	3.2±0.7	3.3±0.7	4.0±0.8	4.6±0.5	4.4±0.5	1.4±0.5	3.2±0.8	4.0±0.7	3.4±0.5	3.7±0.8	2.6±0.5	3.2±0.8	3.6±1.7	3.2±0.4	2.4±0.5	3.4±0.5	2.8±0.4	2.8±0.8	3.4±0.5	3.6±0.5	3.6±0.5	3.8±0.4
Light-K97	3.0±0.0	2.2±0.6	3.5±0.5	3.0±0.0	2.2±0.4	2.4±0.5	2.8±0.8	3.4±0.5	2.1±0.9	2.3±0.7	2.6±0.5	2.2±0.4	2.0±0.7	2.6±0.9	2.2±0.8	3.2±0.4	3.0±0.7	2.2±0.8	3.0±0.0	3.4±0.5	2.8±0.4	2.6±0.5
Light-K97-DH	3.0±0.5	3.6±0.5	3.2±0.7	3.6±0.5	2.9±0.5	2.8±0.8	3.4±0.9	3.4±0.9	3.0±0.6	3.4±0.9	3.0±0.0	3.0±0.7	2.6±0.9	3.2±0.4	2.2±0.8	3.8±0.4	2.6±0.5	2.4±0.9	3.2±0.5	3.6±0.5	3.5±0.5	3.4±0.5
Light-S33	3.2±0.6	2.6±0.7	3.2±0.8	3.0±0.7	2.6±0.5	3.4±0.5	2.6±0.5	3.6±0.5	3.0±0.7	2.6±0.5	2.4±0.5	2.4±0.5	2.2±0.4	2.8±0.8	2.2±0.8	3.6±0.5	2.6±0.5	2.4±0.5	3.0±0.4	2.8±0.8	3.2±0.4	3.4±0.5
Light-S33-DH	3.0±0.8	4.2±0.6	3.1±0.7	4.2±0.8	3.7±0.4	1.4±0.5	3.6±0.5	3.4±0.9	3.0±0.0	3.5±0.5	2.6±0.5	2.8±0.8	3.3±0.8	3.0±0.0	2.1±0.9	3.4±0.5	2.6±0.5	2.6±0.8	3.4±0.4	4.0±0.7	3.4±0.9	3.4±0.5
Very Light-M21	2.8±0.8	4.4±0.5	3.3±0.4	3.4±0.5	3.6±0.5	2.9±0.5	4.2±0.4	4.2±0.4	2.8±0.4	3.4±0.5	2.2±0.4	3.0±0.7	2.6±0.5	2.4±0.5	2.6±0.5	3.0±0.7	3.2±0.8	2.4±0.9	3.2±0.5	3.6±0.5	3.2±0.6	3.6±0.9
Very Light-M21-DH	2.4±0.6	4.6±0.5	3.4±0.5	4.5±0.5	4.2±0.4	3.7±0.9	3.4±0.5	2.3±0.7	2.2±0.4	2.2±0.4	1.7±0.4	1.7±0.4	2.1±0.9	2.5±1.2	2.2±0.8	3.2±0.7	3.4±0.8	3.0±1.2	2.8±0.4	4.4±0.5	3.4±0.4	2.2±0.8
Very Light-K97	3.0±0.0	4.4±0.8	3.8±0.5	2.8±0.8	2.6±0.8	3.5±0.7	3.2±0.5	2.6±0.9	2.4±0.8	2.3±0.6	2.3±0.7	2.3±0.7	2.0±0.7	2.9±0.9	2.6±0.8	2.9±0.2	2.8±0.5	2.6±0.7	2.8±0.2	3.4±0.8	3.2±0.7	2.8±0.8
Very Light-K97-DH	3.0±0.0	3.8±0.8	3.7±0.4	3.8±0.4	3.8±0.4	3.8±0.6	3.4±0.5	3.0±0.8	2.6±0.5	2.7±0.7	2.3±0.7	2.5±0.7	2.3±0.4	2.5±0.5	2.4±0.8	3.5±0.5	3.0±0.0	2.4±0.9	3.2±0.4	3.8±0.4	3.2±0.5	3.6±0.9
Very Light-S33	2.8±0.4	3.4±0.5	3.9±0.7	3.2±0.8	3.0±0.7	3.6±0.4	3.6±0.9	3.1±0.5	2.0±0.6	2.0±0.7	2.2±0.8	3.9±0.2	2.2±0.8	2.0±0.7	2.2±0.9	3.0±0.7	3.2±0.8	2.4±0.9	2.9±0.4	3.4±0.9	3.0±0.7	2.6±0.9

Very Light-S33-DH	2.8±0.4	3.8±0.6	3.4±0.5	4.4±0.5	3.8±0.0	3.2±0.9	3.8±0.4	4.0±0.7	2.9±0.7	2.9±0.7	2.5±0.5	2.8±0.8	2.7±0.7	2.8±0.8	2.6±0.5	3.6±0.5	3.0±0.7	2.6±0.7	2.8±0.4	3.6±0.5	3.6±0.4	4.0±1.0
Ultra Light-M21	2.9±0.5	3.5±0.5	3.7±0.5	3.5±0.5	3.2±0.4	3.1±0.4	3.6±0.4	3.4±0.4	2.7±0.8	2.8±0.4	2.4±0.4	2.9±0.5	2.5±0.4	2.7±0.4	2.3±0.7	3.3±0.0	3.1±0.0	2.8±0.9	3.0±0.4	3.7±0.4	3.4±0.0	3.7±0.7
Ultra Light-M21-DH	3.0±0.0	3.8±0.4	3.1±0.5	4.8±0.4	3.8±0.4	2.4±0.4	4.0±0.0	3.8±0.8	3.3±0.8	3.9±0.2	2.8±0.4	2.3±0.4	2.5±0.5	3.5±0.5	2.3±0.8	3.3±0.4	3.0±0.0	2.5±0.9	3.3±0.4	3.8±0.4	3.3±0.4	4.0±0.7
Ultra Light-S33	3.0±0.6	2.6±0.4	3.0±0.7	2.3±0.4	2.0±0.0	2.6±0.4	2.5±0.5	2.8±0.8	2.5±0.5	3.0±0.0	2.3±0.4	2.5±0.5	2.0±0.7	2.9±0.5	2.3±9.8	2.8±0.4	2.8±0.4	2.4±0.8	2.8±0.4	3.3±0.4	3.0±0.4	2.8±0.4
Ultra Light-S33-DH	3.0±0.0	3.1±0.5	3.8±0.4	3.9±0.2	3.6±0.4	2.1±0.4	3.5±0.5	3.8±0.4	3.0±0.7	3.5±0.5	2.6±0.4	2.9±0.2	2.3±0.4	3.5±0.5	1.8±0.8	3.3±0.4	3.3±0.4	2.3±0.8	3.3±0.4	3.8±0.4	3.3±0.4	4.0±0.7
